# Identification of Muscidae (Diptera) of medico-legal importance by means of wing measurements

**DOI:** 10.1007/s00436-017-5426-x

**Published:** 2017-03-16

**Authors:** Andrzej Grzywacz, Jakub Ogiela, Adam Tofilski

**Affiliations:** 1 Chair of Ecology and Biogeography, Nicolaus Copernicus University, Lwowska 1, 87-100 Toruń, Poland; 2 Department of Pomology and Apiculture, Agricultural University, 29 Listopada 54, 31-425 Kraków, Poland

**Keywords:** Diptera, Muscidae, Species identification, Forensic entomology, Morphometrics, Wing veins

## Abstract

Cadavers attract numerous species and genera of Muscidae, both regular elements of carrion insect assemblages, and accidental visitors. Identification of adult Muscidae may be considered difficult, particularly by non-experts. Since species identification is a vital first step in the analysis of entomological material in any forensic entomology orientated experiment and real cases, various alternative methods of species identification have been proposed. We investigated possibility of semiautomated identification by means of wing measurements as an alternative for classic morphology and DNA-based approaches. We examined genus-level identification success for 790 specimens representing 13 genera of the most common European cadavers visiting Muscidae. We found 99.8% of examined specimens correctly identified to the genus-level. Without error, the following were identified: *Azelia*, *Eudasyphora*, *Graphomya*, *Hydrotaea*, *Musca*, *Muscina*, *Mydaea*, *Neomyia*, *Polietes*, *Stomoxys* and *Thricops*. Genus-level misidentifications were found only in *Helina* and *Phaonia*. Discrimination of examined material on the species level within *Hydrotaea* (318 specimens representing eight species) and *Muscina* (163 specimens representing four species) showed lower, yet still high average identification success, 97.2 and 98.8%, respectively. Our results revealed relatively high success in both genus and species identification of Muscidae of medico-legal importance. Semiautomated identification by means of wing measurements can be used by non-experts and does not require sophisticated equipment. This method will facilitate the identification of forensically relevant muscids in comparison to more difficult and more time-consuming identification approaches based on taxonomic keys or DNA-based methods. However, for unambiguous identification of some taxa, we recommend complementary use of identification keys.

## Introduction

The Muscidae is a large dipteran family comprised of more than 5000 species. Representatives of the family are widespread throughout all biogeographic regions. Some species have increased their range of distribution due to commerce and currently are considered cosmopolitan (Skidmore 1985). The association between man and ubiquitous flies, inter alia *Musca domestica* Linnaeus and *Musca sorbens* Wiedemann, is traceable to the earliest times of recorded history (Greenberg and Kunich 2002; Schmidt 2006). Even today, some African tribes use houseflies in traditional medicine and in rituals to gain spiritual protection and prosperity (Lawal and Banjo 2007). Muscids are known from a broad range of life strategies, both in immature and adult stages (Skidmore 1985). From the medical and veterinary point of view, the most important are species causing irritation to people and animal due to their numerous occurrence, vectors of pathogenic microorganisms, biting species feeding on blood, and those that reveal parasitic behavior in immature stages. However, in larval stages, muscids can be often found in a variety of decomposing organic matter of animal and plant origin. They can reveal saprophagous or either facultative or obligatory predatory behavior.

Insects’ association with cadavers and their utility for medico-legal purposes has been well known for a long time (Benecke 2001). In forensic practice, the examination of entomological material collected from dead bodies allows one to answer certain questions, which most often is to estimate the minimum time since death (post-mortem interval (PMI)). Animal carrion and dead human bodies are also attractive habitats for many muscid species (Fiedler et al. 2008), and the family is considered as one of the arthropod groups of forensic importance (Byrd and Castner 2010). Recently, Grzywacz et al. (2017) catalogued about 200 muscid taxa associated with carrion and dead human bodies worldwide. However, many of these species are not considered regular elements of carrion community assemblages, but instead, they represent taxa that may occasionally visit cadavers. In forensic entomology, significant conclusions can be made from the analysis of arthropod species composition on the dead body. For this purpose, only species of established forensic usefulness should be taken into the consideration (Matuszewski et al. 2010). Thus, it is necessary to discriminate accurately between species of no forensic usefulness, who are often accidental visitors, and those of established forensic usefulness. Recently, significant progress has been done in the field of the identification of Diptera of medico-legal importance. High-quality and well-illustrated morphological keys facilitate the identification of forensically relevant species (e.g., Rochefort et al. 2015; Akbarzadeh et al. 2015). However, species diversity of non-regular visitors in some cases may exceed the number of species of forensic usefulness (Matuszewski et al. 2011). In case of Muscidae, it is recommended that identification keys to adult flies associated with carrion should cover a wide range of taxa, not only those known from their forensic usefulness (Grzywacz et al. 2016). This raises some issues about the possibility of species identification. Adult Muscidae identification is based mostly on thorax and leg chaetotaxy and wing venation (Gregor et al. 2002) and may be considered difficult. This hinders detailed investigation of their medico-legal usefulness in carrion succession experiments. On the other hand, molecular libraries allowing for species identification by means of DNA barcoding still do not cover the full set of muscid taxa recognized as visiting animal and human cadavers (e.g., Boehme et al. 2012; Renaud et al. 2012).

Similarly to other biological studies, in forensic entomology, species identification is a prerequisite for any further analysis of the collected material (Gotelli 2004). An alternative method of identification may be geometric morphometrics of wing veins. This method allows one to detect subtle differences between studied specimens on various taxonomic levels (Alves et al. 2016). Wing morphometrics has already been shown as a valuable method for the identification of closely related species (Lyra et al. 2010; Van Cann et al. 2015) or populations (Hall et al. 2014) of some medically and veterinary important species. However, previous studies did not attempt to investigate the application of this method on a broad scale that will allow for the identification of certain group of forensically important insects.

The objective of this study was to investigate whether semiautomated identification by means of wing measurement can be complementary and/or surrogate to morphological and DNA-based identification methods for European Muscidae considered forensically important. We aimed to study identification success for two taxonomic levels. Firstly, we checked identification success on the genus level for common cadaver-visiting muscids. Subsequently, we studied species identification success within two significant, for forensic purposes, genera, *Hydrotaea* Robineau-Desvoidy and *Muscina* Robineau-Desvoidy.

## Material and methods

**Fig. 1:**
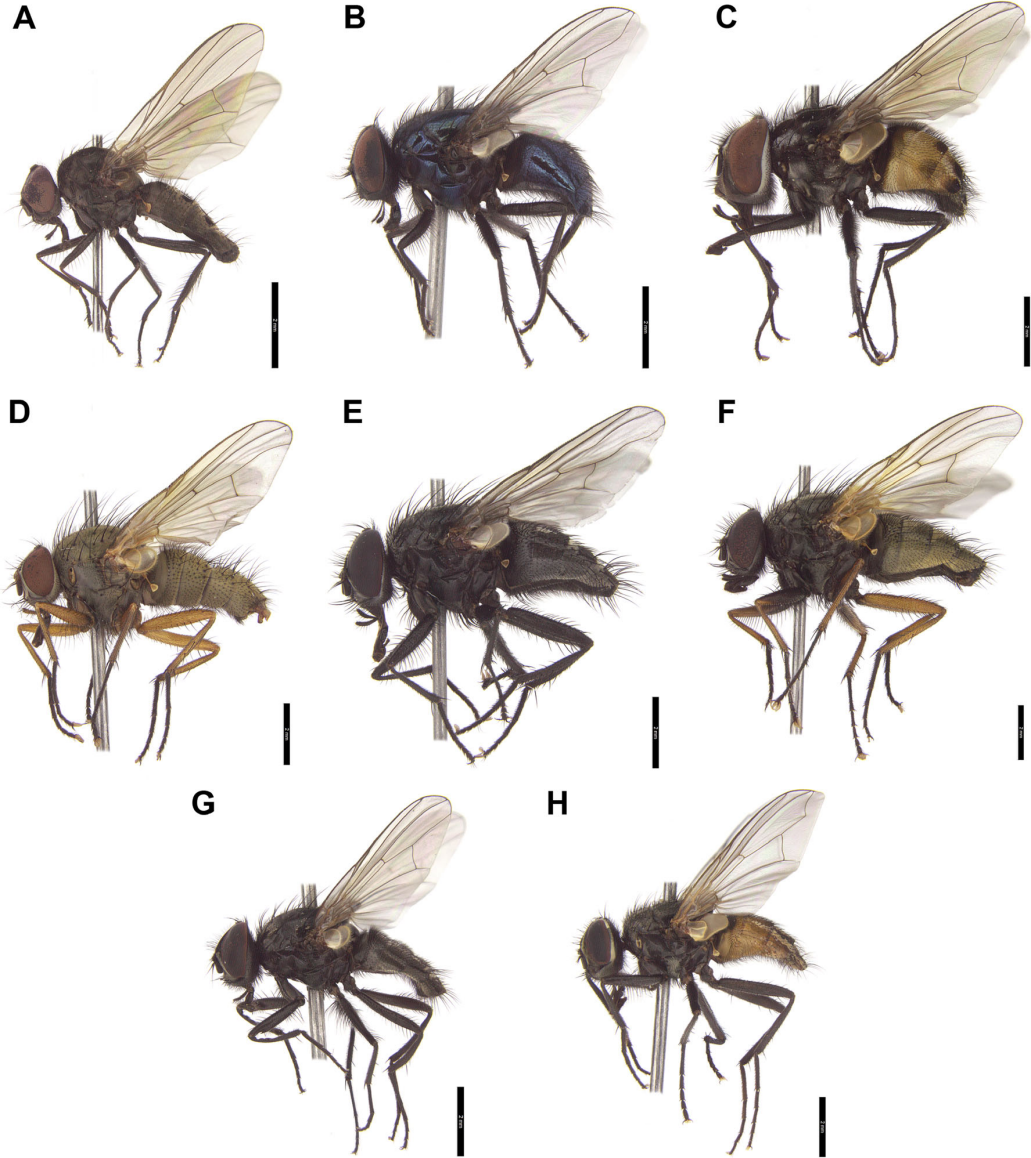
Males of selected muscid species representing genera used in this study. **a** *Azelia nebulosa* Robineau-Desvoidy. **b** *Eudasyphora cyanicolor* (Zetterstedt). **c** *Graphomya maculata* (Scopoli). **d** *Helina impuncta* (Fallén). **e** *Muscina levida* (Harris). **f** *Mydaea urbana* (Meigen). **g** *Hydrotaea dentipes* (Fabricius). **h** *Musca domestica* Linnaeus

**Fig. 2:**
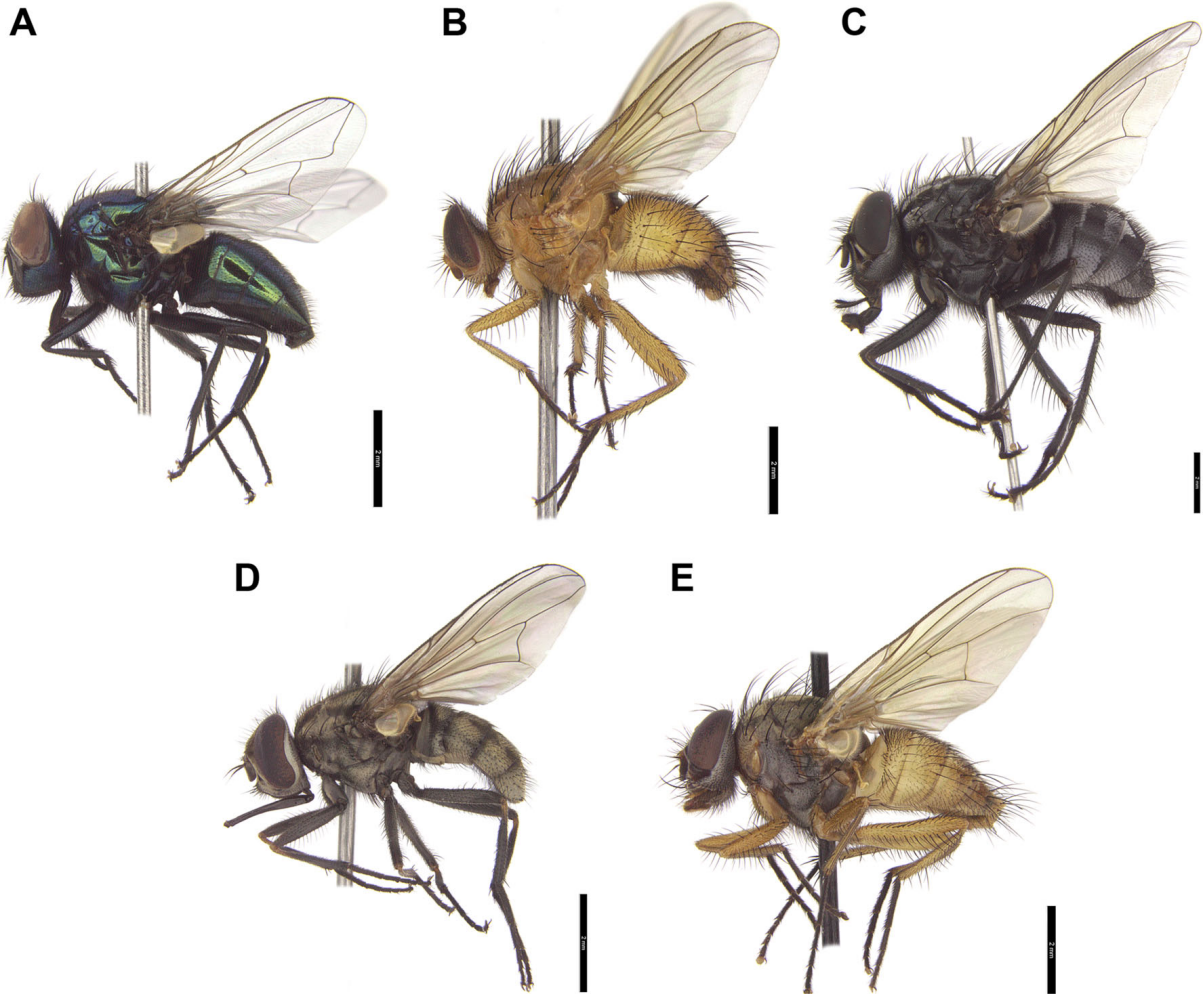
Males of selected muscid species representing genera used in this study. **a** *Neomyia cornicina* (Fabricius). **b** *Phaonia pallida* (Fabricius). **c** *Polietes lardarius* (Fabricius). **d** *Stomoxys calcitrans* Linnaeus. **e** *Thricops simplex* (Wiedemann)

Species and genera for the present study were selected based on the data found in publications. We sampled European muscid genera commonly visiting animal carrion and human bodies. Material for the present study was collected in central and southern Poland. Adult flies, both males and females, were collected directly with an entomological net or lured to slightly decomposed chicken liver. Insects have been identified according to Gregor et al. (2002). To examine genus-level identification success, we studied 790 specimens representing 13 muscid genera (Figs. 1 and 2). Subsequently, we examined species-level identification success for specimens representing eight species of *Hydrotaea* (318 specimens) and four species of *Muscina* (163 specimens). Most of the studied genera were represented by single species.

**Fig. 3:**
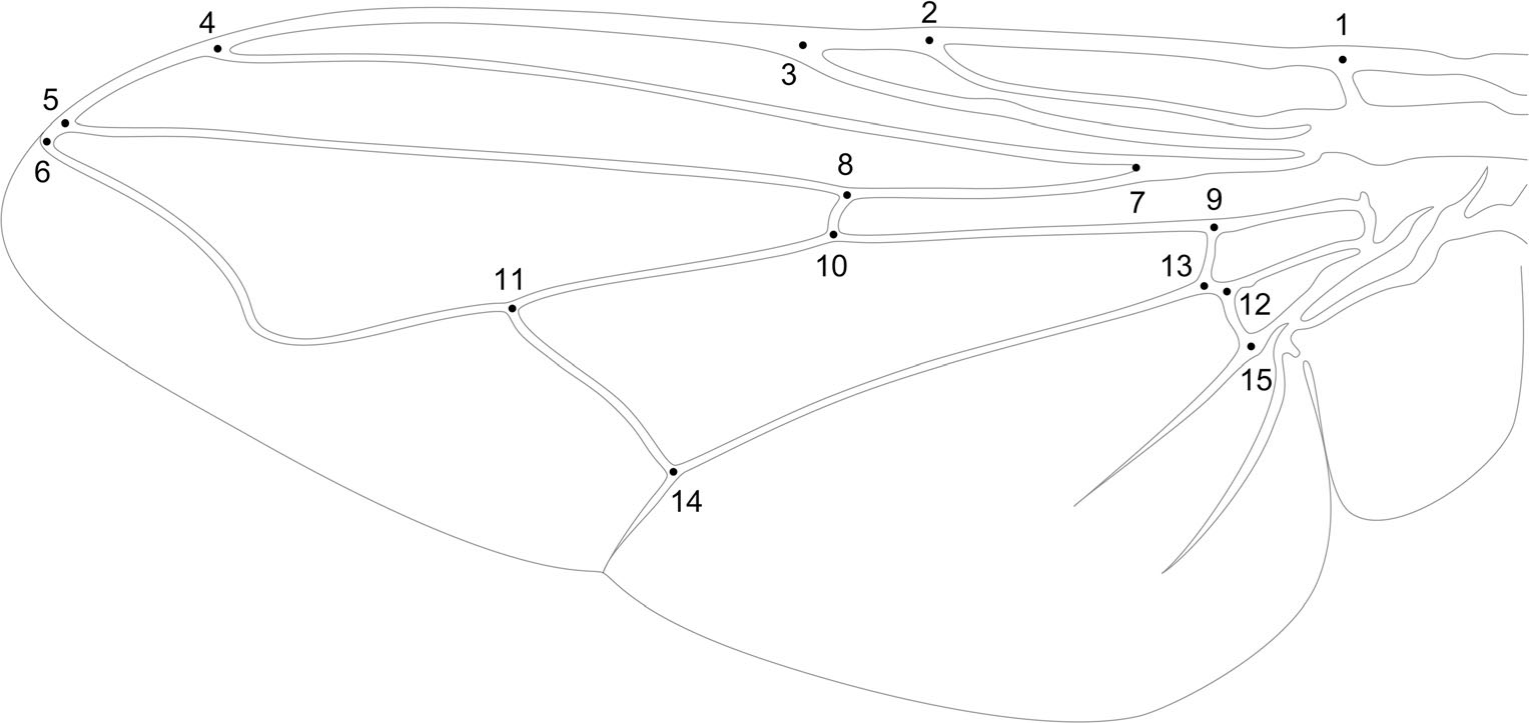
Wing of male of *Musca domestica*. The numbered points indicate the landmarks used for wing measurements

Both wings have been detached from the body and flattened under microscopic glass. Wing images were obtained using a camera (UCMOS09000KPB, ToupTek Photonics) equipped with a 25-mm lens (FL-CC2514-2M, Ricoh). Resolution of the images was 2841 pixels per centimeter. On the wing images, 15 homologous landmarks (Fig. 3) were determined in IdentiFly software (Tofilski 2008; Przybyłowicz et al. 2016). The software was also used for the implementation of the species identification algorithm. It can be downloaded from http://www.drawwing.org/identifly.

Both left and right wings were measured, and the mean value of the two measurements was used in the statistical analysis. The coordinates of the landmarks were analyzed using methods of geometric morphometrics. For landmark superimposition, we used a generalized orthogonal least-squares procedure (Rohlf and Slice 1990) in MorphoJ software (Klingenberg 2011). Only wing shape and not wing size was used in the analysis. The wing shape was described by coordinates of the aligned landmarks, and it was compared between species and genera using multivariate analysis of variance (MANOVA) in Statistica (StatSoft Inc 2014). Identification of species and genera was based on the canonical variate analysis (CVA) of wing shape. The identification was validated using the leave-one-out method in PAST 3.14 software (Hammer et al. 2001).

## Results

**Fig. 4:**
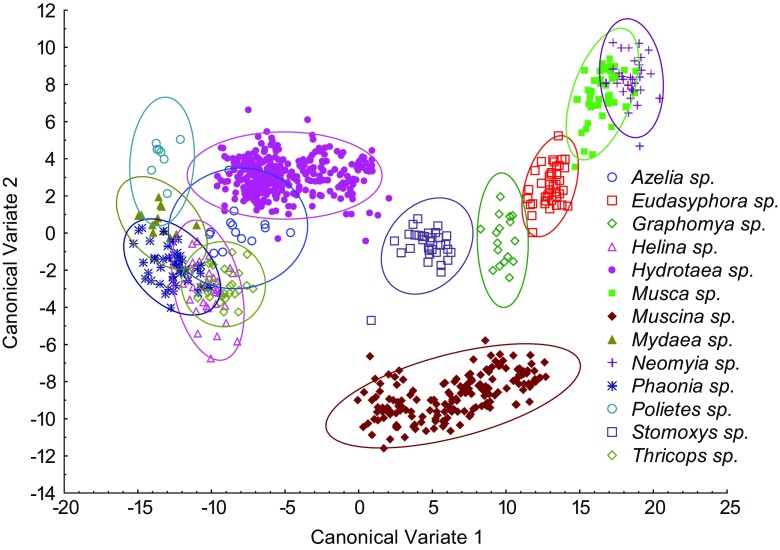
Discrimination of Muscidae genera based on canonical variate analysis

The wing shape differed markedly between genera (MANOVA Wilks’ lambda <0.0001, *P* < 0.0001) and species (MANOVA Wilks’ lambda <0.0001, *P* < 0.0001). The differences allowed for the discrimination of examined taxa with very high identification success, both on the genus and species levels. Canonical variate analysis revealed most genera forming robust, well-differentiated clusters of points (Fig. 4). In the graph of the first two canonical variates (Fig. 4), there is overlap between some of the clusters; however, most of them were well separated in other dimensions, which are not shown. In consequence, identification success estimated with cross-validation allowed us to correctly identify 99.8% of examined specimens to the genus level (Table 1). The only genus-level misidentifications occurred in representatives of *Helina* Robineau-Desvoidy and *Phaonia* Robineau-Desvoidy. One specimen of *Helina* (2.9%) was misidentified as *Phaonia*, and one specimen of *Phaonia* (2.2%) was incorrectly classified as *Mydaea* Robineau-Desvoidy (Table 1).

**Table 1:** Identification error of animal and human bodies visiting muscid genera assessed using leave-one-out cross - validation

	Classified as														
	*Azelia* sp.	*Eudasyphora* sp.	*Graphomya* sp.	*Helina* sp.	*Hydrotaea* sp.	*Musca* sp.	*Muscina* sp.	*Mydaea* sp.	*Neomyia* sp.	*Phaonia* sp.	*Polietes* sp.	*Stomoxys* sp.	*Thricops* sp.	Total	Correct identifications (%)
*Azelia* sp.	14	–	–	–	–	–	–	–	–	–	–	–	–	14	100
*Eudasyphora* sp.	–	40	–	–	–	–	–	–	–	–	–	–	–	40	100
*Graphomya* sp.	–	–	17	–	–	–	–	–	–	–	–	–	–	17	100
*Helina* sp.	–	–	–	34	–	–	–	–	–	*1*	–	–	–	35	97.1
*Hydrotaea* sp.	–	–	–	–	318	–	–	–	–	–	–	–	–	318	100
*Musca* sp.	–	–	–	–	–	46	–	–	–	–	–	–	–	46	100
*Muscina* sp.	–	–	–	–	–	–	163	–	–	–	–	–	–	163	100
*Mydaea* sp.	–	–	–	–	–	–	–	14	–	–	–	–	–	14	100
*Neomyia* sp.	–	–	–	–	–	–	–	–	30	–	–	–	–	30	100
*Phaonia* sp.	–	–	–	–	–	–	–	*1*	–	44	–	–	–	45	97.8
*Polietes* sp.	–	–	–	–	–	–	–	–	–	–	8	–	–	8	100
*Stomoxys* sp.	–	–	–	–	–	–	–	–	–	–	–	32	–	32	100
*Thricops* sp.	–	–	–	–	–	–	–	–	–	–	–	–	28	28	100

Rows represent a given genus and columns represent a predicted genus. Correct identifications are in the diagonal and incorrect are in italic

**Fig. 5:**
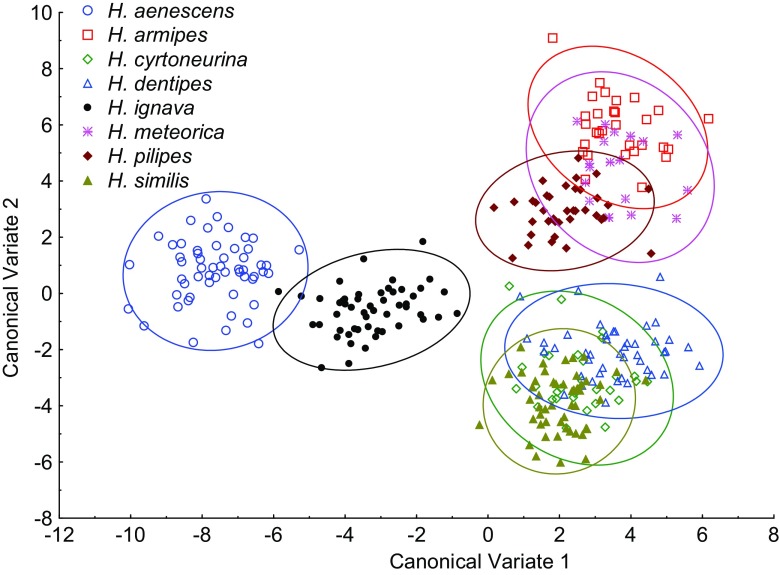
Discrimination of eight species of the *Hydrotaea *based on canonical variate analysis

All representatives of *Hydrotaea* and *Muscina* were correctly identified to the genus level. Subsequent analyses on the species level within both genera also revealed a relatively high identification success, 97.2 and 98.8%, respectively. Erroneous identifications in *Hydrotaea* occurred in nine cases from 318 examined specimens. The misidentifications occurred between closely related species (Table 2). Single representative of *Hydrotaea armipes* (Fallén) (3.3%) was erroneously identified as *Hydrotaea meteorica* (Linnaeus), and a single specimen of the latter (5.6%) was assigned to *Hydrotaea pilipes* Stein species. All remaining misidentifications within *Hydrotaea* were found within the *dentipes* species group. Two specimens of *Hydrotaea cyrtoneurina* (Zetterstedt) (6.7%) were determined erroneously as *Hydrotaea dentipes* (Fabricius). Three representatives of *H. dentipes* (6.8%) were erroneously identified, one as *H. cyrtoneurina* and two as *Hydrotaea similis* Meade. Two representatives of the latter species (3.9%) were misidentified as *H. dentipes*. Canonical analysis of the first two variates showed *Hydrotaea* species forming three groups of clusters (Fig. 5). *Hydrotaea aenescens* (Wiedemann) and *Hydrotaea ignava* (Harris) formed a group of points well differentiated from the remaining *Hydrotaea*. The second group is comprised of representatives of the aforementioned *dentipes* species group and the third group of *H. armipes*, *H. meteorica*, and *H. pilipes*.

**Table 2:** Identification error of animal and human bodies visiting species of *Hydrotaea* assessed using leave-one-out cross-validation

	Classified as									
	*Hydrotaea aenescens*	*Hydrotaea armipes*	*Hydrotaea cyrtoneurina*	*Hydrotaea dentipes*	*Hydrotaea ignava*	*Hydrotaea meteorica*	*Hydrotaea pilipes*	*Hydrotaea similis*	Total	Correct identifications (%)
*Hydrotaea aenescens*	55	–	–	–	–	–	–	–	55	100
*Hydrotaea armipes*	–	29	–	–	–	*1*	–	–	30	96.7
*Hydrotaea cyrtoneurina*	–	–	28	*2*	–	–	–	–	30	93.3
*Hydrotaea dentipes*	–	–	*1*	41	–	–	–	*2*	44	93.2
*Hydrotaea ignava*	–	–	–	–	52	–	–	–	52	100
*Hydrotaea meteorica*	–	–	–	–	–	17	*1*	–	18	94.4
*Hydrotaea pilipes*	–	–	–	–	–	–	37	–	37	100
*Hydrotaea similis*	–	–	–	*2*	–	–	–	50	52	97.2

Rows represent a given genus and columns represent a predicted genus. Correct identifications are in the diagonal and incorrect are in italic

**Fig. 6:**
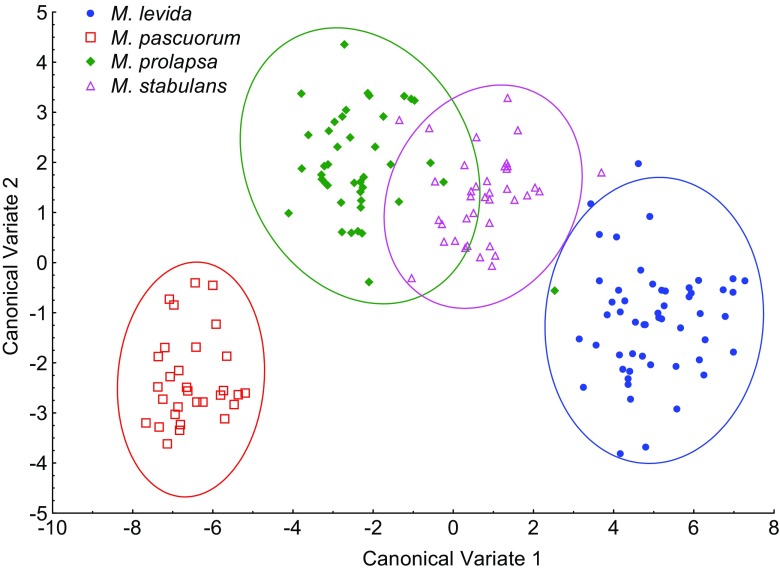
Discrimination of four species of the *Muscina *based on canonical variate analysis

Representatives of the four studied *Muscina* species formed well-defined clusters of points after the canonical variates analysis, and only *Muscina prolapsa* (Harris) and *Muscina stabulans* (Fallén) partly overlapped each other (Fig. 6). Misidentifications within *Muscina* were observed in two representatives of *M. prolapsa* (4.8%), which were erroneously determined as *Muscina levida* (Harris) and *M. stabulans*, respectively (Table 3).

**Table 3:** Identification error of animal and human bodies visiting species of *Muscina* assessed using leave-one-out cross-validation

	Classified as					
	*Muscina levida*	*Muscina pasquorum*	*Muscina prolapsa*	*Muscina stabulans*	Total	Correct identifications (%)
*Muscina levida*	53	–	–	–	53	100
*Muscina pasquorum*	–	30	–	–	30	100
*Muscina prolapsa*	*1*	–	40	*1*	42	95.2
*Muscina stabulans*	–	–	–	38	38	100

Rows represent a given genus and columns represent a predicted genus. Correct identifications are in the diagonal and incorrect are in italic

## Discussion

The data presented here show that wing measurements can be useful for the identification of Muscidae. This confirms earlier studies concerning other insects (Gaston and O’Neill 2004). In Diptera, the wing measurements were used successfully for the quantification of both within and between species variations (Brown 1980; Klingenberg et al. 1998; Alves and Bélo 2002; Hall et al. 2014; Siomava et al. 2016). The wing venation differs markedly between Diptera species, and it can be used for identification of mosquitoes (Dujardin 2011; Sumruayphol et al. 2016), tephritid flies (Van Cann et al. 2015), tsetse flies (Kaba et al. 2016), screwworm flies (Lyra et al. 2010), and stable flies (Changbunjong et al. 2016). Our study is the first extensive attempt to investigate the usefulness of wing measurements for the identification of dipterans reported from animal carrion and dead human bodies.

Identification of adult Muscidae may be considered difficult, particularly by non-experts without training and access to the reference collection. Probably due to problems with identification, some researchers did not attempt to identify muscids collected in carrion succession experiments or muscids were referred to at the genus or family level only (e.g., Wolff et al. 2001; Martinez et al. 2007; Segura et al. 2009; Bygarski and Leblanc 2013). We have found that wing venation analysis has great potential for the identification of Muscidae. We have observed a very high identification success rate, both at the genus and species levels (Tables 1, 2, and 3). Wing measurements proved to be particularly suitable for discrimination between muscid genera of established forensic importance (*Musca* Linnaeus, *Hydrotaea*, *Muscina*) and non-regular carrion visitors (*Azelia* Robineau-Desvoidy, *Eudasyphora* Townsend, *Graphomya* Robineau-Desvoidy, *Mydaea*, *Neomyia* Walker, *Thricops* Rondani). Most species from the latter group were reported from cadavers only from single or very few specimens (Grzywacz et al. 2017). However, for example, *Thricops* may be present on carrion, in some cases, in large numbers (Matuszewski et al. 2008). These non-regular elements of carrion fauna in most cases were properly identified in our analysis. Misidentifications were observed only within *Helina* and *Phaonia*, which are considered as closely related genera (Kutty et al. 2014; Haseyama et al. 2015). If reported, *Helina* and *Phaonia* were represented by single or very few specimens, and none of them has been considered a regular element of carrion fauna (Grzywacz et al. 2017). In immature stages, these genera are obligatory predators living in humus soil, animal dung, or under tree trunks (Skidmore 1985). Non-regular elements of carrion fauna are supposed to be present on cadavers to feed on fluids coming from the decomposing cadaver, if the opportunity occurs and no significant conclusions can be drawn from the analysis of their residency patterns. Thus, the first step in the analysis of entomological material for medico-legal purposes is to discriminate them from species of forensic usefulness. This can be difficult, because the random carrion visitors represent many diverse taxa, and in some cases, they can be misidentified as species of forensic importance (Grzywacz et al. 2016). Wing measurements can minimize such risk, since all random carrion visitors in this study were discriminated from forensically important genera (Fig. 4 and Table 1).

In forensic entomology literature, *Hydrotaea* and *Muscina* are among the most often referred to muscid genera (Grzywacz et al. 2017). Both genera formed very well separated clusters of points (Fig. 4), and they were discriminated without errors (Table 1). According to our data, they can be unambiguously identified by means of wing measurements. Wing measurements can be particularly useful for the identification of females of *Hydrotaea*. These muscids cause severe problems for non-experts because of chaetotaxy details used in the identification keys (Gregor et al. 2002). Within both *Hydrotaea* and *Muscina*, we have observed 97.2 and 98.8% average species identification success, respectively. In *Hydrotaea*, we found three clusters of species (Fig. 5): (1) *ignava* group (*H. ignava* and *H. aenescens*), (2) *dentipes* group (*H. cyrtoneurina*, *H. dentipes*, *H. similis*), and (3) remaining species (*H. armipes*, *H. meteorica*, *H. pilipes*). This corresponds with classification proposed by Skidmore (1985) after examination of larval morphology: (1) genus *Ophyra*, (2) *Hydrotaea* subgenus *Hydrotaeoides*, and (3) *Hydrotaea* s. str., respectively. In this work, we consider *Ophyra* Robineau-Desvoidy as a junior synonym of *Hydrotaea* (Savage and Wheeler 2004; Grzywacz unpublished). We found that all specimens of *Hydrotaea aenescens* and *H. ignava* were correctly identified. In forensic entomology literature, these two species are the most often referred to representatives of *Hydrotaea* (in Grzywacz et al. 2017). Identification of remaining species has not been without errors, yet misidentifications have always been restricted to closely related species (Table 2).

Our results revealed relatively high success in both genus and species identification of Muscidae. This makes semiautomated identification by means of the wing geometric morphometric method a promising tool for species identification of European carrion visiting Muscidae. This approach is a low cost, relatively easy method, which does not require sophisticated equipment. In comparison to DNA barcoding, this method is much cheaper and faster. Wings for the analysis can be collected from freshly preserved specimens or long-dead specimens as long as the wings are not damaged and all necessary landmarks can be marked on a wing (Lyra et al. 2010). Major advantages of the method are the ease and short time of procedure. Analysis can be done by non-experts and requires only very basic training. Insect wing detached from the body after short preparation requires digitalization. Subsequently, certain landmarks must be marked in a proper order using IdentiFly software, which is freely available at http://www.drawwing.org/identifly. Together with the software, we provide all necessary information for the identification of the species used in this study. However, the method of identification of Muscidae described here should be used with care, because misidentifications are possible. Moreover, the present study covers only a limited number of European taxa. It is recommended to verify the identification using traditional identification keys (e.g., Gregor et al. 2002). An important advantage of the method is quantifying similarity of a specimen to a range of species and genera. Particular attention should be paid to outliers that show low similarity to all taxa or high similarity to more than one taxon. Such non-typical specimens should be examined more carefully or sent for verification to a specialist.

## Conclusions

Despite the fact that many muscid species and genera were reported from animal carrion and dead human bodies, only some of them were recognized as useful for medico-legal purposes. We found that wing measurements allow for precise identification of forensically relevant muscid genera. Particularly, we found a very high success rate for identification of regular vs. random elements of carrion fauna. Although we observed very high identification success for many species of *Hydrotaea* and *Muscina*, for unambiguous identification of examined material, we recommend complementary use of identification keys to discriminate between closely related species to avoid possible misidentifications.

## References

[CR1] Akbarzadeh K, Wallman JF, Suláková H, Szpila K (2015). Species identification of middle eastern blowflies (Diptera: Calliphoridae) of forensic importance. Parasitol Res.

[CR2] Alves SM, Bélo M (2002). Morphometric variations in the housefly, *Musca domestica* (L.) with latitude. Genetica.

[CR3] Alves VM, Moura MO, de Carvalho CJB (2016) Wing shape is influenced by environmental variability in *Polietina orbitalis* (Stein) (Diptera: Muscidae). Rev Bras Entomol 60:150–156. doi:10.1016/j.rbe.2016.02.003

[CR4] Benecke M (2001). A brief history of forensic entomology. Forensic Sci Int.

[CR5] Boehme P, Amendt J, Zehner R (2012). The use of COI barcodes for molecular identification of forensically important fly species in Germany. Parasitol Res.

[CR6] Brown KR (1980). Comparative wing morphometrics of some calyptrate Diptera. Aust J Entomol.

[CR7] Bygarski K, Leblanc HN (2013). Decomposition and arthropod succession in Whitehorse, Yukon territory, Canada. J Forensic Sci.

[CR8] Byrd JH, Castner JL, Byrd JH, Castner JL (2010). Insects of forensic importance. Forensic entomology: the utility of arthropods in legal investigations.

[CR9] Changbunjong T, Sumruayphol S, Weluwanarak T et al (2016) Landmark and outline-based geometric morphometrics analysis of three *Stomoxys *flies (Diptera: Muscidae). Folia Parasitol (Praha). doi:10.14411/fp.2016.037 10.14411/fp.2016.037 27827335

[CR10] Dujardin J-P (2011) Modern morphometrics of medically important insects. In: Tibayrenc M (ed) Genetics and Evolution of Infectious Disease. Elsevier, pp 473–501

[CR11] Fiedler A, Halbach M, Sinclair B, Benecke M (2008). What is the edge of a forest? A diversity analysis of adult Diptera found on decomposing piglets inside and on the edge of a western German woodland inspired by a courtroom question. Entomol heute.

[CR12] Gaston KJ, O’Neill MA (2004). Automated species identification: why not?. Philos Trans R Soc Lond Ser B Biol Sci.

[CR13] Gotelli NJ (2004). A taxonomic wish-list for community ecology. Philos Trans R Soc Lond Ser B Biol Sci.

[CR14] Greenberg B, Kunich JC (2002). Entomology and the law: flies as forensic indicators.

[CR15] Gregor F, Rozkošný R, Barták M, Vaňhara J (2002) The Muscidae (Diptera) of central Europe. Folia Fac Sci Nat Univ Masaryk Brun Biol 107:1–280

[CR16] Grzywacz A, Amendt J, Fremdt H (2016). Seek, and ye shall find—the example of *Neohydrotaea lundbecki* (Michelsen) (Diptera: Muscidae), a rare muscid species or just ignored so far in forensic entomology?. North West J Zool.

[CR17] Grzywacz A, Hall MJR, Pape T, Szpila K (2017). Muscidae (Diptera) of forensic importance—an identification key to third instar larvae of the western Palaearctic region and a catalogue of the muscid carrion community. Int J Legal Med.

[CR18] Hall MJR, MacLeod N, Wardhana AH (2014). Use of wing morphometrics to identify populations of the old world screwworm fly, *Chrysomya bezziana* (Diptera: Calliphoridae): a preliminary study of the utility of museum specimens. Acta Trop.

[CR19] Hammer Ø, Harper DAT, Ryan PD (2001). PAST: paleontological statistics software package for education and data analysis. Paleaontologia Electron.

[CR20] Haseyama KLF, Wiegmann BM, Almeida EAB, de Carvalho CJB (2015). Say goodbye to tribes in the new house fly classification: a new molecular phylogenetic analysis and an updated biogeographical narrative for the Muscidae (Diptera). Mol Phylogenet Evol.

[CR21] Kaba D, Berte D, Ta Bi Tra D (2016). The wing venation patterns to identify single tsetse flies. Infect Genet Evol.

[CR22] Klingenberg CP (2011). MorphoJ: an integrated software package for geometric morphometrics. Mol Ecol Resour.

[CR23] Klingenberg CP, McIntyre GS, Zaklan SD (1998). Left-right asymmetry of fly wings and the evolution of body axes. Proc Biol Sci.

[CR24] Kutty SN, Pont AC, Meier R, Pape T (2014). Complete tribal sampling reveals basal split in Muscidae (Diptera), confirms saprophagy as ancestral feeding mode, and reveals an evolutionary correlation between instar numbers and carnivory. Mol Phylogenet Evol.

[CR25] Lawal OA, Banjo AD (2007). Survey for the usage of arthropods in traditional medicine in southwestern Nigeria. J Entomol.

[CR26] Lyra MLL, Hatadani LMM, de Azeredo-Espin AMLML, Klaczko LBB (2010). Wing morphometry as a tool for correct identification of primary and secondary new world screwworm fly. Bull Entomol Res.

[CR27] Martinez E, Duque P, Wolff M (2007). Succession pattern of carrion-feeding insects in Paramo, Colombia. Forensic Sci Int.

[CR28] Matuszewski S, Bajerlein D, Konwerski S, Szpila K (2010). Insect succession and carrion decomposition in selected forests of central Europe. Part 2: composition and residency patterns of carrion fauna. Forensic Sci Int.

[CR29] Matuszewski S, Bajerlein D, Konwerski S, Szpila K (2011). Insect succession and carrion decomposition in selected forests of central Europe. Part 3: succession of carrion fauna. Forensic Sci Int.

[CR30] Matuszewski S, Bajerlein D, Konwerski S, Szpila K (2008). An initial study of insect succession and carrion decomposition in various forest habitats of central Europe. Forensic Sci Int.

[CR31] Przybyłowicz Ł, Pniak M, Tofilski A (2016). Semiautomated identification of European corn borer (Lepidoptera: Crambidae). J Econ Entomol.

[CR32] Renaud AK, Savage J, Adamowicz SJ (2012). DNA barcoding of northern Nearctic Muscidae (Diptera) reveals high correspondence between morphological and molecular species limits. BMC Ecol.

[CR33] Rochefort S, Giroux M, Savage J, Wheeler TA (2015). Key to forensically important Piophilidae (Diptera) in the Nearctic region. Can J Arthropod Identif.

[CR34] Rohlf FJ, Slice D (1990). Extensions of the procrustes method for the optimal superimposition of landmarks. Syst Zool.

[CR35] Savage J, Wheeler TA (2004). Phylogeny of the Azeliini (Diptera: Muscidae). Stud Dipterologica.

[CR36] Schmidt E (2006). Remains of fly puparia as indicators of Neolithic cattle farming. Environ Archaeol.

[CR37] Segura NA, Usaquen W, Sanchez MC (2009). Succession pattern of cadaverous entomofauna in a semi-rural area of Bogota, Colombia. Forensic Sci Int.

[CR38] Siomava N, Wimmer EA, Posnien N (2016). Size relationships of different body parts in the three dipteran species *Drosophila melanogaster*, *Ceratitis capitata* and *Musca domestica*. Dev Genes Evol.

[CR39] Skidmore P (1985). The biology of the Muscidae of the world. Ser Entomol.

[CR40] Sumruayphol S, Apiwathnasorn C, Ruangsittichai J (2016). DNA barcoding and wing morphometrics to distinguish three Aedes vectors in Thailand. Acta Trop.

[CR41] Tofilski A (2008). Using geometric morphometrics and standard morphometry to discriminate three honeybee subspecies. Apidologie.

[CR42] Van Cann J, Virgilio M, Jordaens K, De Meye M (2015). Wing morphometrics as a possible tool for the diagnosis of the *Ceratitis fasciventris*, *C. anonae*, *C. rosa* Complex (Diptera, Tephritidae). Zookeys.

[CR43] Wolff M, Uribe A, Ortiz A, Duque P (2001). A preliminary study of forensic entomology in Medellin, Colombia. Forensic Sci Int.

